# Concise Review: Adipose-Derived Stem Cells (ASCs) and Adipocyte-Secreted Exosomal microRNA (A-SE-miR) Modulate Cancer Growth and proMote Wound Repair

**DOI:** 10.3390/jcm8060855

**Published:** 2019-06-15

**Authors:** Pietro Gentile, Simone Garcovich

**Affiliations:** 1 Surgical Science Department, Plastic and Reconstructive Surgery Unit, University of “Tor Vergata”, 00133 Rome, Italy; 2 Institute of Dermatology, F. Policlinico Gemelli IRCSS, Università Cattolica del Sacro Cuore, 00168 Rome, Italy; simgarko@yahoo.it

**Keywords:** cancer inhibition, metastasis, microenvironment, adipose-derived stem cells, ASC-based drug delivery therapeutic systems, oncological safety, wound repair

## Abstract

Adipose-derived stem cells (ASCs) have been routinely used from several years in regenerative surgery without any definitive statement about their potential pro-oncogenic or anti-oncogenic role. ASCs has proven to favor tumor progression in several experimental cancer models, playing a central role in regulating tumor invasiveness and metastatic potential through several mechanisms, such as the paracrine release of exosomes containing pro-oncogenic molecules and the induction of epithelial-mesenchymal transition. However, the high secretory activity and the preferential tumor-targeting make also ASCs a potentially suitable vehicle for delivery of new anti-cancer molecules in tumor microenvironment. Nanotechnologies, viral vectors, drug-loaded exosomes, and micro-RNAs (MiR) represent additional new tools that can be applied for cell-mediated drug delivery in a tumor microenvironment. Recent studies revealed that the MiR play important roles in paracrine actions on adipose-resident macrophages, and their dysregulation has been implicated in the pathogenesis of obesity, diabetes, and diabetic complications as wounds. Numerous MiR are present in adipose tissues, actively participating in the regulation of adipogenesis, adipokine secretion, inflammation, and inter-cellular communications in the local tissues. These results provide important insights into Adipocyte-secreted exosomal microRNA (A-SE-MiR) function and they suggest evaluating the potential role of A-SE-MiR in tumor progression, the mechanisms underlying ASCs-cancer cell interplay and clinical safety of ASCs-based therapies.

## 1. Introduction

A clinical need exists for the development of cellular therapy to improve the regeneration of damaged tissues. A great variety of tissues would benefit from tissue engineering-based repair, such as cutaneous and sub-cutaneous, skin, cartilage, bone, hard and soft tissues. The increase of complexity in the targeted tissue for repair, necessitate a concomitant increase in the complexity of the related tissue engineering approach. Because of the complexity of the targeted tissue, tissue-engineering strategies involve the use of cells and biologically active factors to improve new tissue generation. This strategy can involve de novo growth by ex vivo and in vitro culture or by in vivo regeneration. In the last decade, many researchers have shown the clinical implications of Mesenchymal Stem Cells (MSCs) such as adipose-derived stem cells (ASCs) in damaged tissue. The Mesenchymal and Tissue Stem Cell Committee of the International Society for Cellular Therapy (ISCT) has proposed a minimal set of four criteria to define MSCs [1]: 

1. MSCs are plastic-adherent when maintained under standard culture conditions. 

2. MSCs have the capability to differentiate in vitro in osteoblasts, adipocytes, chondroblasts (as demonstrated by staining of in vitro cell culture) [1]. 

3. MSCs express CD73, CD90, and CD105. 

4. MSCs lack expression of the hematopoietic lineage markers c-kit, CD14, CD11b, CD34, CD45, CD19, CD79, and human leukocyte antigen-DR. 

The ASCs meet the majority of the ISCT’s criteria for MSCs [1].

As is commonly known, fat tissue is a multifunctional organ that contains various cellular types, such as the stromal vascular fraction (SVF) and mature adipocytes.

The SVF provides a rich source of ASCs that can be easily isolated from human fat [2,3,4].

Therefore, the ASCs are routinely used from several years in regenerative surgery without however any definitive statement about their potential pro-oncogenic or anti-oncogenic role.

ASCs has proven to favor tumor growth in several experimental cancer models, playing a central role in regulating tumor invasiveness and metastatic potential through several mechanisms, such as the paracrine release of exosomes containing pro-oncogenic molecules and the induction of epithelial-mesenchymal transition [2,4,5,6,7,8,9,10,11].

However, the high secretory activity and the preferential tumor targeting make also ASCs a potentially suitable vehicle for delivery of new anti-cancer molecules in tumor microenvironment [12,13,14,15,16,17,18,19,20,21,22,23,24,25].

In the recent years, it has become progressively more evident that tumor-cell features are as much important as their interaction with the microenvironment [2]. The latter is characterized by a complex interplay between different cellular types coordinated through a composite signaling network [3]. These interactions are able to influence tumor invasive capacity and its metastatic potential [4]. MSCs are part of this complex system together with immune cells, adipocytes, myofibroblasts, extracellular matrix and tumor cells [26]. The most studied MSCs are cord blood mesenchymal stem cells (CB-MSCs), bone-marrow mesenchymal stem cells (BM-MSCs) peripheral blood stem cells (PB-SCs) and ASCs [27].

Over the years, ASC-based therapies have been tested and used in many clinical settings both oncological and non-oncological like inflammatory bowel disease, chronic ischemic cardiomyopathy, rheumatoid arthritis, soft-tissue sarcoma reconstruction, graft-versus-host disease, outcomes of breast cancer and breast reconstruction [28,29,30,31].

Parallel to the diffusion of ASCs as a possible therapeutic agent in many medical conditions, a growing concern has been raised about their possible pro-oncogenic risk.

In this review we discussed the role of ASCs, adipokines, chemokines and Adipocyte-secreted exosomal microRNA (A-SE-miR) in the inhibition of cancer growth, to promote the wound repair.

## 2. Implications of ASCs and Their Adipokines and Chemokines in Cancer Growth

### 2.1. Adipokines, Obesity and Breast Cancer Modulation

The role that is played by the fat tissue and their secretions like the adipokines is beginning to be recognized in cancer growth modulation. Plasma adipokine levels, which are modulated during obesity, could have effects on breast carcinogenesis [31]. Growing evidence has therefore recognized obesity as a main oncological risk factor and peri-tumor fat tissue as well as its progenitor cells as a source of pro-tumor factors [5]. At the same time, the relationship between ASCs and cancer has been deeply investigated using pre-clinical models [32]. Although in numerous clinical studies ASCs use did not appear to increase the risk of loco-regional or distant tumor recurrence, it has not been provided yet a strong definitive evidence on its oncological safety. Breast cancer cells (BCCs) are surrounded and locally influenced by an adipocyte microenvironment, which is probably more extensive in obese people [31]. In a study of Strong et al. [33], leptin appears to be strongly involved in mammary carcinogenesis and it may contribute to the local pro-inflammatory mechanisms, especially in obese patients, who have increased metastatic potential and greater risk of mortality. On this field, Gentile et al. [31] reported the percentage of recurrences in three different group of patients in which the study group was treated with fat injection enriched with ASCs for breast reconstruction, control group 1 was treated with a fat injection that was not enriched and control group 2 was not treated. In a group of seven patients (CG2) (all affected by obesity), three recurrences (two systemic and one local) were recorded, as compared with four recurrences (three systemic and one local) in a study group (SG) that was included 121 patients and five recurrences (two systemic and three local), while control group 1 (CG1) was composed by 50 patients [31]. Strong et al. [33] reported that ASCs that were derived from the abdominal subcutaneous adipose tissue of obese subjects (BMI > 30) enhanced BCCs proliferation in vitro and tumorigenicity in vivo. These findings were correlated with changes in the gene expression profile of BCCs after co-culturing with ASCs, particularly in the estrogen receptor-alpha (ESR1) and progesterone receptor (PGR) expression [33]. An analysis of the gene expression profile of the four groups of ASCs revealed obesity induced alterations in several key genes, including leptin (LEP). Blocking estrogen signaling with ICI182.780, leptin neutralizing antibody or letrozole diminished the impact of ASCs that are derived from obese subjects [33]. Women that were diagnosed with estrogen receptor/progesterone receptor positive (ER+/PR+) breast cancers (BC) that also expressed high levels of leptin had poorer prognosis than women with low leptin expression [33]. The results from the study of Strong et al. [33] demonstrate that abdominal obesity induces significant changes in the biological properties of ASCs and that these alterations enhance ER+/PR+ BC tumorigenesis through estrogen dependent pathways.

### 2.2. Chemokines and Growth Factors Role in Breast Cancer Modulation

ASCs can be involved in the promotion of tumor growth, invasiveness and metastatic potential by several pathways. Their pro-angiogenic capacity though the secretion of growth factors (GFs) and chemokines such as vascular endothelial growth factors (VEGF), platelet-derived growth factors (PDGF) c-kit induce proliferation of endothelial cells and development of a tumor-supporting vascular network [6,7,34]. Moreover, ASCs have immune-modulating proprieties mediated by transforming growth factor-β1 (TGF-β1), hepatocyte growth factors (HGF) and interferon-γ (INF-γ) impairing immune-mediated response to tumor [7,12,35,36,37,38,39]. They are also able to induce drug resistance and cell proliferation in the BCCs line MCF-7/ADR (a multidrug-resistant BCCs model) mediated through C-terminal Src kinase (Csk)-binding protein (Cbp) expression [8].

Epithelial to mesenchymal transition (EMT) has been highlighted as a fundamental passage in tumor history and in its shift toward a more invasive and metastatic phenotype [40]. It has been reported that ASCs can induce EMT in BCCs by acting on multiple pathways, especially through PI3K/AKT signaling and p38 MAP kinase [41,42] or by overexpressing leptin, as shown by ASCs from obese patients [33]. ASCs transformation, through the inhibition of Wnt signaling, into tumor-associated fibroblasts by breast tumor-derived factors has been also reported [43]. The same myofibroblastic differentiation is reported in ASCs exposed to BC exosomes through the induction of TGF-β signaling [44]. ASCs themselves are able to secrete exosomes that induce BCCs migration mediated by Wnt-signaling [45].

### 2.3. Relationship between ASCs and Cancer Cells

ASCs contribution to a neoplastic microenvironment does not seem to be limited to cells located in its immediate vicinity. In a mouse cancer model, tumor was able to recruit ASCs from distant fat tissue sites through the systemic circulation, promoting tumor growth [46]. Cancer cells (CCs) together with its stroma and inflammatory cells secrete several factors such as MCP-1 and SDF-1 that induce ASCs homing and migration to tumor microenvironment [47]. 

An increased number of circulating ASCs have been demonstrated in obese patients with a history of colorectal cancer, prostate cancer and BC although the fate of these cells and the clinical significance of this finding have not been clarified yet [9,48,49].

In a mouse tumor model, a comparison between lean and obese mice revealed a six-fold higher concentration of ASCs in systemic circulation of the obese one. Those circulating ASCs were localized in the tumor stroma [50].

On the other hand, tumor-homing properties of ASCs could be also exploited in a therapeutic way by transforming ASCs in vehicles to deliver anti-neoplastic agents directly inside a cancer microenvironment. MSCs have been tested as vectors for several innovative cancer therapies such as drug-loaded nanoparticles, micro-RNAs, viral vectors encoding tumor suppressor genes and many others [51].

ASCs present numerous advantages compared to other MSCs. Their harvesting is less invasive with a cell yield more than 1000-fold higher when compared to BM-MSCs and CB-MSCs [10,52]. Furthermore, they have a longer life-span, higher proliferative capacity, shorter doubling time and later in vitro senescence compared to BM-MSCs [53]. These features have to be considered when choosing an optimal cellular carrier for therapies on a wide scale. Simultaneous presence of noxious and beneficial aspects proper of ASC-cancer interplay earned ASCs the definition of “double-edged sword” [11,13].

## 3. An Unexpected Effect from Adipocyte-Secreted Exosomal microRNA 

### 3.1. ASCs-Exosomes Role in Cancer Growth and Wound Repair

Exosomes (intraluminal vesicles < 100 nm) are secreted by cells working as intercellular transmitters of mRNA, microRNA (miR), and proteins [14]. The importance of ASC-secreted exosomes (A-SE) in promoting wound repair has been recently reported [54]. It has been hypothesized that exosomes from ASCs could be internalized by dermal fibroblasts stimulating their migration, proliferation, and collagen synthesis [54]. A study by Seo et al. reports the ability of miR-503-3pf, released by A-SE, to inhibit tumor growth regulating cancer stem cell (CSCs) proliferation and self-renewal, reducing the expression of pluripotency genes. Moreover, xenograft tumor growth is inhibited by the administration of miR-503-3p, supporting this miR as a stemness-attenuating factor via cell-to-cell communications [15]. In rats with N1S1-induced hepatocellular carcinoma (HCC), the administration of A-SE induces significantly smaller tumors and volume ratios, more circulating and intra-tumoral natural killer T (NKT) cells, and low-grade HCC compared to untreated controls, sustaining the hypothesis that A-SE can promote NKT-cell antitumor response and induce HCC suppression and low-grade tumor differentiation [55]. Exosomes from ASCs conditioned medium inhibit proliferation, wound-repair and colony formation ability as reported in A2780 and SKOV-3 ovarian cancer cells [16]. A-SE induced apoptosis signaling through the up-regulation of different pro-apoptotic genes, such as BAX, CASP9, and CASP3, while down-regulating the anti-apoptotic BCL2. In fact, by sequencing exosomal RNAs, a rich population of miRNAs with anti-cancer activities has been identified [16]. On metastatic prostate cancer (PCa), ASC-derived conditioned medium inhibits tumor proliferation and induce apoptosis both in vitro and in vivo [56]. MiR array analysis on A-SE shows the up-regulation of different miRs; among them, miR-145, known as a tumor suppressor, has been identified. The knockdown of miR-145 in ASCs reverts the anti-tumoral effects, while also reducing the expression of caspase 3/7 and increasing the anti-apoptotic protein BclxL [57]. However, some authors reported the pro-tumoral activity of A-SE, promoting BC cell migration through Wnt signaling [45].

Contrasting data from literature report that ASCs can affect glioma and glioblastoma growth [17,18,19,58]. In particular, the conditioned medium from ASCs seems to promote the epithelial-to-mesenchymal-like transition in glioma cells in vitro [58]. In addition, ASCs are reported to increase glioblastoma cell migration that displays a strong tropism on ASCs [17], likely due to their chemokine secretion that mediates cell migration [18]. Meanwhile, Yang et al. demonstrated an induction of apoptosis and differentiation in U251 glioma cell line by ASCs conditioned medium [19]. However, some researchers reported that exosomes from ASCs conditioned medium are ineffective on U87MG glioblastoma cells, even if their internalization into tumor cells occurs [20].

### 3.2. ASCs-microRNA Relationship with Cancer Cells 

A possible explanation for these conflicting results arise from a multiplicity of factors influencing the interaction between ASCs and CCs, such as their origin and pre-treatments, cancer type and different in vitro and in vivo conditions (e.g., ASCs/CCs ratio, cell injection modality, kinetics of carcinogenesis) that may affect the experimental standardization [12]. However, as reported above, it’s a common belief that several secreted factors, produced by cancer and inflammatory cells, induce the homing and migration of ASCs into the tumor microenvironment [47].

ASCs represent the best candidates for exosome-wrapped miR strategy, as they can release large amounts of exosomes [21]. Since glioma cells and glioma stem cells (GSCs), a small subpopulation of cancer stem cells implicated in therapeutic resistance and tumor recurrence, express very low levels of miR-124 and miR-145, Lee et al. successfully tried to deliver, through A-SE, these miR mimics in glioma cells and GSC co-cultures. The internalization, via gap junction-dependent and independent processes, determines a decrease in their respected target genes, SCP-1 and Sox2, reducing glioma cell migration and GSC self-renewal. Moreover, when administered intracranially, ASCs are able to deliver miR-124 mimic to glioma xenografts [22].

As reported by Lou et al., ASCs transfected with a miR-122 expression plasmid are able to deliver miR-122 through their exosomes affecting cell viability, apoptosis, and cell cycle of hepatocellular carcinoma (HCC) cells. In addition, ASCs transfected with miR-122 also sensitize HCC xenograft to sorafenib in vivo. It is known that HCC displays a high resistance to conventional chemotherapy and miR-122 is proven to be essential to promote chemosensitivity, representing a valid tool for a targeted strategy [23].

It has been also reported the pro-apoptotic activity of NK cell-differentiated ASCs transfected with miR-150 on pancreatic cancer cells PANC1 [24]. miR-150 is responsible for the development and activation of NK cells as well as their production of IFNγ, and this strategy shows an effective immunomodulatory activity [24]. CSCs are a small population with stem cell-like properties found in tumors that influences tumor progress, metastasis, and drug resistance. In a study by Lee et al. the authors hypothesize an anti-cancer therapy based on CSC reprogramming into non-tumorigenic cells using A-SE. Briefly, exosomes from osteogenic differentiated human ASCs, containing specific cargos with osteoinductive properties, successfully induce CSCs to express osteogenic-related genes, such as alkaline phosphatase, osteocalcin, and runt-related transcription factor 2. In addition, the differentiation decreases some drug-resistance genes such as ATP binding cassette transporter, the breast cancer gene family (BCRA1 and BCRA2), and the ErbB gene family [25].

## 4. Promotion of Wound Healing

### 4.1. Wound Healing Process

As is known, the wound healing process consists of three major overlapping stages: (1) an inflammatory stage; (2) a proliferative stage and (3) a resolution stage.

The inflammatory stage is triggered by an initiating pathogen or toxin that results in the release of pathogen- or damage-associated molecular patterns (PAMPs or DAMPs, respectively) that ligate and activate pattern recognition receptors such as toll-like receptors (TLRs), NOD-like receptors (NLRs), C-type lectin receptors (CLRs) or other receptors on resident cells, including endothelial cells, mast cells, tissue macrophages and interstitial fibroblasts [59,60]. Receptor activation triggers the production and secretion of cytokines, chemokines and growth factors that induce inflammation and the recruitment of inflammatory cells, primarily neutrophils and monocytes [61]. The recruited monocytes are pro-inflammatory and will subsequently differentiate into inflammatory (M1) macrophages. The activated resident cells and the recruited inflammatory (M1) macrophages release toxic ROS that destroy invading pathogens [62,63] and induce the expression of genes encoding various cytokines, inflammatory molecules and multiple proteases including MMPs, serine and cysteine proteases, and elastases.

Several mechanisms are involved in these effects, including alterations of the balance between pro-inflammatory and anti-inflammatory cytokine secretion [64,65,66,67,68,69], down-regulation of pro-inflammatory transcription factors important for neutrophil survival such as NF-κB and IRF1 [70,71], and concomitant up-regulation of the anti- inflammatory transcription factor IRF-4 [72,73]. These effects collectively promote inflammatory (M1) to wound healing/pro-fibrotic (M2) macrophage phenotype transition and initiate the resolution of inflammation [64,65,66,67,68,69].

The transition from an inflammatory phenotype (M1) to a wound healing/profibrotic (M2) phenotype induces the progression from the inflammation phase to the tissue repair phase.

Monocytes/macrophages (MM) at the transition between the inflammatory and tissue repair/wound healing stages produce copious amounts of cytokines and growth factors that promote the proliferation of multiple cell types involved in damaged tissue repair [74,75,76,77,78].

Wound healing/profibrotic (M2) macrophages seem at this stage, either via differentiation of fresh recruited infiltrating monocytes or by in place transition of antecedently differentiated infiltrating inflammatory (M1) macrophages to a wound healing/profibrotic (M2) phenotype. STAT6 is activated during this transition and promotes IL-4/IL-13-mediated differentiation of wound healing/profibrotic (M2) macrophages by up-regulating their expression of arginase (Arg1) and multiple other wound healing/profibrotic phenotype genes [79]. Wound healing/profibrotic macrophages possess associate degree medicinal drug makeup and stimulate and activate fibroblasts towards their overstated ECM production and secretion [74,75,76,77,78,79].

The macrophage phenotype is also influenced by changes in the mechanical, cellular and metabolic characteristics of the target tissue [80,81,82].

In the final phase, the wound scar tissue is remodeled by replacement of the provisional ECM with a stronger, durable ECM, characterized by extensive collagen cross-linking and the gradual replacement of type III collagen with type I collagen [83,84,85].

These changes are followed by senescence or apoptosis of activated myofibroblasts [86,87] and regression of the neo-vascularization [88,89,90].

### 4.2. ASCs Relationship with Regeneration Process

The bio-molecular mechanisms of ASCs and their products involved in tumor growth have been analyzed so far. In light of the information previously reported, it remains to be clarified which is, indeed, the bio-molecular mechanism involving ASCs and their products in the tissue regeneration process. To better understand these pathways, it may be useful to report in this section the pathophysiological similarities between the processes of regeneration and growth of the tumor and the participation in them of the ASCs.

For this reason, the tumor could be defined as "the wound that never heals".

In fact, analogies between the molecular mechanisms of ASC homing to the tumor tissue are detected with the molecular homing mechanisms of ASCs in the damaged area during the different phases of tissue regeneration process. Furthermore, analogies are found between the molecular mechanisms of ASCs involvement in angiogenesis and tumor vasculogenesis and in the regeneration zone.

As reported in a study by Akama et al. [91] they found that IL6 mediates the expression of Mmp13 and Timp1 in visceral fat derived-ASCs and the TCF21-dependent expression of Mmp2 and Col4a1 is IL6-independent. The basic helix-loop-helix transcriptional regulator, transcription factor 21 (TCF21), is a marker gene for white adipose tissues and is abundantly expressed in visceral-derived ASCs. These results suggest that TCF21 contributes to the pro-inflammatory environment in visceral fat depots and to active extracellular matrix (ECM) remodeling of these depots by regulating IL6 expression and MMP-dependent ECM remodeling in a spatiotemporally coordinated manner.

In the resolution stage, the wound scar tissue shows a more durable ECM, characterized by extensive collagen cross-linking followed by regression of the neo-vascularization [88,89,90]. As a consequence of the bio-molecular pathway involved in the wound healing promotion by ASCs, more clinical studies reported the clinical efficacy of ASCs in scar treatment [92], in breast reconstruction [3,31,93,94,95,96,97] and in chronic wounds [97,98,99,100,101,102].

Now it seems necessary, however, to clarify the bio-molecular mechanisms through which the ASCs promote tissue healing.

ASCs are recognized to promote wound healing of otherwise chronic wounds, possibly through the reduction of inflammation, induction of angiogenesis, and promotion of fibroblast and keratinocyte growth [103]. However, little is known regarding the importance of ASC-produced ECM for wound healing. Among the resident skin cells that express integrins—and thus may be subjected to modulation by the ECM—are fibroblasts and keratinocytes [104]. In addition, proteins in the ECM modulate the activity of growth factors and cytokines such as PDGF and TGF-β, produced by activated platelets and macrophages, respectively [105,106]. Thus, the ECM functions as a reservoir by protecting the growth factors from degradation and controlling their release [107].

In the chronic wound environment, in vitro and in vivo studies suggest that the ASCs may be able to discontinue the prolonged inflammation phase and restore the progression through the phases of proliferation and remodeling. In terms of effects on the inflammatory processes, it is well known that ASCs may induce a conversion of the macrophage phenotype from the pro-inflammatory M1 associated with chronic wounds to the anti-inflammatory and wound healing M2 phenotype [108,109]. During the proliferation phase, secreted factors from ASCs enhance several fibroblast characteristics, such as cell proliferation, migration and, importantly, the synthesis of collagen and other ECM proteins [110,111,112]. Furthermore, ASCs have been demonstrated to inhibit ECM degradation through the increased binding of matrix metalloproteinases (MMPs) and secretion of tissue inhibitors of metalloproteinases (TIMPs) [113]. The ability of ASCs to promote new vessel growth is therefore relevant to wound healing [114]. Finally, in vitro studies suggest that ASCs may promote re-epithelialization through modulation of keratinocytes in terms of promoting their proliferation and migration, but more studies are needed to confirm if this also holds true for chronic wounds [115,116].

## 5. Concluding Remarks

An explanation for the conflicting data reported indicating that ASCs can show pro-oncogenic and/or anti-oncogenic role arise from a multiplicity of factors influencing the interaction between ASCs and CCs, such as their origin and pre-treatments, cancer type and different experimental conditions that may affect standardization. Appropriate models considering not only CCs but also the surrounding microenvironment should be developed for this purpose.

On the other hand, tumor-homing properties of ASCs could be also exploited in a therapeutic way. ASCs could be vehicles to deliver anti-neoplastic agents directly inside cancer microenvironment. For this reason, ASCs have been tested in pre-clinical models as vectors for several innovative cancer therapies such as drug-loaded exoxomes and nanoparticles, micro-RNAs, viral vectors encoding tumor suppressor genes and many others. 

Clinical data reported allow us to make a clear statement about safety of ASC use in regenerative surgery. More robust evidence is needed to clarify the pro-oncological and anti-oncological role of ASCs in order to fully exploit their encouraging potential in a drug-delivery system.
